# A New Route of Fucoidan Immobilization on Low Density Polyethylene and Its Blood Compatibility and Anticoagulation Activity

**DOI:** 10.3390/ijms17060908

**Published:** 2016-06-09

**Authors:** Kadir Ozaltin, Marián Lehocký, Petr Humpolíček, Jana Pelková, Petr Sáha

**Affiliations:** 1 Centre of Polymer Systems, Tomas Bata University in Zlín, Tr. Tomase Bati 5678, 760 01 Zlín, Czech Republic; kadirozaltin@hotmail.com (K.O.); humpolicek@ft.utb.cz (P.H.); saha@rektorat.utb.cz (P.S.); 2 Department of Hematology, Vsetin Hospital, Nemocnicni 955, 755 01 Vsetin, Czech Republic; pelkova@fhs.utb.cz; 3 Faculty of Humanities, Tomas Bata University in Zlín, Mostni 5139, 760 01 Zlín, Czech Republic

**Keywords:** biomaterials, fucoidan, heparin, thrombosis, anticoagulant, plasma treatment

## Abstract

Beside biomaterials’ bulk properties, their surface properties are equally important to control interfacial biocompatibility. However, due to the inadequate interaction with tissue, they may cause foreign body reaction. Moreover, surface induced thrombosis can occur when biomaterials are used for blood containing applications. Surface modification of the biomaterials can bring enhanced surface properties in biomedical applications. Sulfated polysaccharide coatings can be used to avoid surface induced thrombosis which may cause vascular occlusion (blocking the blood flow by blood clot), which results in serious health problems. Naturally occurring heparin is one of the sulfated polysaccharides most commonly used as an anticoagulant, but its long term usage causes hemorrhage. Marine sourced sulfated polysaccharide fucoidan is an alternative anticoagulant without the hemorrhage drawback. Heparin and fucoidan immobilization onto a low density polyethylene surface after functionalization by plasma has been studied. Surface energy was demonstrated by water contact angle test and chemical characterizations were carried out by Fourier transform infrared spectroscopy and X-ray photoelectron spectroscopy. Surface morphology was monitored by scanning electron microscope and atomic force microscope. Finally, their anticoagulation activity was examined for prothrombin time (PT), activated partial thromboplastin time (aPTT), and thrombin time (TT).

## 1. Introduction

Biocompatibility of polymeric biomaterials can be considered mostly by means of their bulk and surface properties. Mechanical compatibility is important for sufficient stability and appropriate rigidity within bulk properties, whereas material surface compatibility is important to obtain a good interfacial biocompatibility. When a biomaterial is placed into the body, firstly its surface comes into contact with physiological fluids, thus the first interaction is strongly dependent on the surface properties.

When a synthetic biomaterial, with the purpose of implantation or tissue replacement, is introduced into a living biological system it can cause a foreign body reaction and surface induced thrombosis since it is not biologically active as living tissues. The first response of the biological system is the rapid protein adsorption (within seconds) onto a biomaterial's surface, in accordance with the Vroman Effect [1,2]. As such, this response becomes recognizable by the integrin receptors of most of the cells [3]. Thus, cellular interaction with the adsorbed protein layer plays a paramount importance [3,4,5,6,7]. The type, concentration, and conformation of the proteins are important by means of further cellular interactions at the interface [2].

In terms of blood compatibility, the blood response strongly depends on materials’ surface properties, such as surface chemistry, energy, charge density, and wettability [4]. Protein adsorption is followed by platelet adhesion and aggregation, activation of intrinsic pathway of blood coagulation via blood protein factor XII (mostly activated by negatively charged surfaces [8]), fibrin network formation, complement system activation with interactions of erythrocytes and leucocytes [3,5,9,10,11,12]. Therefore, this coagulation cascade triggers the thrombus (blood clot) formation on the biomaterials’ surface, namely surface induced thrombosis [4,5], which may cause blood vessel occlusion or heart attack in the case of its vascular implantation [13,14]. Thus, reducing protein adsorption and platelet activity increase the hemocompatibility of the biomaterial.

Some of the polymeric materials (such as polyolefins) have low surface energy and lack adequate enough bonding interfaces which makes them challenging substrates for further surface modification; as such, surface treatment is essential [15,16]. Functionalization of biomaterials by means of surface treatment is possible by several methods, such as corona discharge, UV irradiation, wet chemistry, flame treatment, ozone induced treatment, flame treatment, and plasma treatment [17,18,19]. Among them, plasma treatment is an effective way to modify polymer surface homogeneously without toxic residues. The heat free process of plasma treatment is another important advantage, especially for heat sensitive polymeric materials, in order to keep their desired bulk properties. Altering the plasma parameters, such as diversity of used gas with its flow rate, reactor type and pressure, generated power, and frequency creates different types of plasma for individual applications like sterilization, deposition, chain scission, ion etching, cross-linking, polymerization, surface activation, *etc.* [15,17,20]. Controlling the process efficiency is possible by changing the exposure time. Plasma treatment can introduce oxygen containing functional groups (such as hydroxyl, carboxyl, carbonyl) onto the polymer surface by means of excited atoms, ions, electrons, neutral species, and ultraviolet light; in doing so, polymer surfaces can become more convenient for further chemical modifications [17,21,22,23].

**Figure 1:**
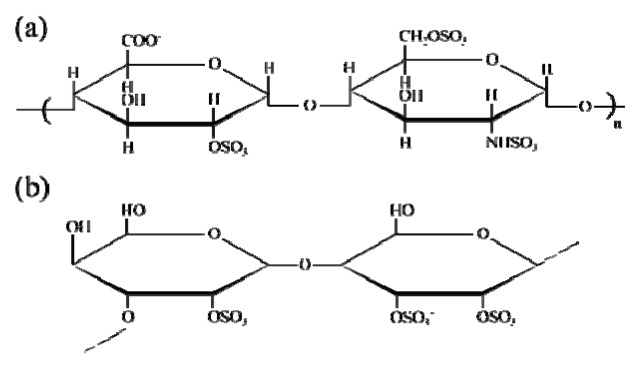
Chemical structure of anticoagulants: (**a**) Heparin; (**b**) Fucoidan.

To avoid blood coagulation, fibrinolysis occurs by a normal body process [24] as a result of the breakdown of blood clots (primary fibrinolysis) or by medical supply (secondary fibrinolysis). Thrombus inhibition by anticoagulant fucoidan is a promising strategy to avoid thrombus formation. Heparin is another polysaccharide (see Figure 1a) that has been used as an anticoagulant for several years [15,25,26]. The biggest drawback of heparin is hemorrhaging and thrombocytopenia [25,26,27]. It may also cause virus-based infections due to the fact that it is mostly obtained from animals [25].

Fucoidan is another sulfated polysaccharide that has attracted scientific interest in recent years as an alternative anticoagulant to heparin. Fucoidan is a marine sourced biopolymer largely found in the intercellular matrix of brown algae [25] and rather limitedly found in marine invertebrates [27,28,29,30] (see Figure 1b). Besides its anticoagulant activity, it has an assortment of biological activities, such as antivirus, anticancer, antitumor, anti-inflammatory, and antioxidant activities. These properties make fucoidan an attractive polysaccharide for numerous biomedical applications [28,29,30,31,32,33,34,35].

Dissimilarly to other polysaccharides, the mechanism of anticoagulant activity of fucoidan is related to the interactions with the natural thrombin inhibitors of antithrombin (AT III) and heparin cofactor II (HCII), activated factor II (thrombin), and activated factor X [26,28,36,37]. The effect of the anticoagulant activity of fucoidan depends on its structural properties, such as sulfation pattern and degree, monosaccharide composition and especially its molecular weight (MW) [27,28,36,38]. For instance, low molecular weight (LMW) fucoidan has been found to be effective for its anti-inflammatory response, while middle and high molecular weight fucoidan has been found to be more effective for its anticoagulant activity, which is related to the altering of sulfate groups by changing MW in order to control the binding properties [27,28,34,36]. Besides the drawbacks of heparin, it has been found that the anticoagulant activity of fucoidan is greater than heparin [39]. On the other hand, the structure of fucoidan is not well defined yet, so as such its applications are presently rather limited.

In this study, low density polyethylene (LDPE) was used as a substrate to functionalize its surface by means of plasma treatment and further polymerization by grafting polymer brushes of Allylamine (AAM), *N*-allylmethylamine (MAAM), and *N*,*N*-dimethylallylamine (DAAM) by introducing an amine functional group, one methyl, and two methyl groups to reveal the optimum polymer brush for the immobilization of the anticoagulants heparin and fucoidan. Surface characterizations were carried out by means of scanning electron microscope (SEM), atomic force microscope (AFM), X-ray photoelectron spectroscopy (XPS), Fourier transform infrared spectroscopy (FTIR), and wettability test. Anticoagulation activity was examined for prothrombin time (PT), activated partial thromboplastin time (aPTT), and thrombin time (TT).

## 2. Results

Due to the similar and unsatisfying anticoagulation behaviors of AAM, MAAM, and DAAM immobilized samples, their investigations, except the anticoagulation test, are not placed in this paper. In fact, as was observed after surface examinations, the existence of their polymer brushes was revealed, however, they did not show any significant effect on further heparin/fucoidan immobilization.

### 2.1. Surface Wettability Behaviour

Surface wettability was carried out by static contact angle measurement by sessile drop technique using distilled water as a testing liquid; results are provided in Table 1. Changes in the water contact angle (θ_w_) by spreading the water on the surface, as a result of bonding interactions of the water molecules, refers to hydrophilicity, which has a correlation with its surface energy. The water contact angle of the reference polyethylene (PE) is drastically decreased by about 33% after plasma treatment (from 85.3° to 56.9°) as a result of the presence of plasma induced hydrophilic oxidative functional groups [40] and also with respect to the surface roughening which is discussed in Section 2.3. Thus, wettability and surface energy is increased and therefore the PET surface becomes more likely to interact with further modifications. It has also been known that such hydrophilic surfaces have significant influence on the cell and blood plasma protein interactions.

Heparin and fucoidan immobilized samples before plasma treatment (PEH and PEF, respectively) and heparin immobilized sample after plasma treatment (PETH) showed almost the same wettability properties (Table 1) due to insufficient immobilization of anticoagulants (see Section 2.2). The fucoidan immobilized sample after plasma treatment (PETF) showed the lowest water contact angle value among all anticoagulant immobilized samples, which corresponds to its hydrophilic character (Table 1). This indicates that fucoidan was successfully immobilized onto the surface, which was also demonstrated by scanning electron microscopy.

### 2.2. Scanning Electron Microscopy Characterization

**Figure 2:**
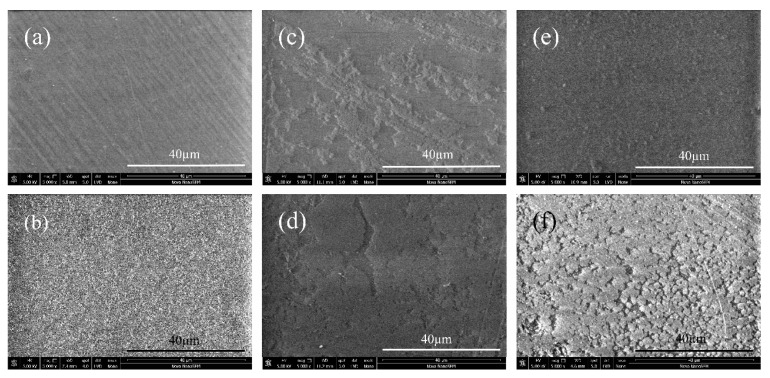
Surface morphology by scanning electron microscope (SEM): (**a**) PE; (**b**) PET; (**c**) PEH; (**d**) PEF; (**e**) PETH; and (**f**) PETF.

Surface morphological images of the samples taken by SEM are shown in Figure 2. The reference PE sample exhibits homogenous, relatively smooth surface morphology with a minor uniform fiber-like feature stem from the production (Figure 2a). After plasma treatment, the surface morphology became rougher due to the surface reorganization by exposition in plasma (Figure 2b). As a result, increased surface area was generated by increasing roughness, which is a desired surface condition for further interactions. Without plasma treatment the immobilization of the anticoagulants onto PE surface were limited since partially attachment was observed (see Figure 2c,d). Therefore, beside immobilized anticoagulants, blood may also contact with the substrate, which results in decrease of antithrombotic effect. Plasma modified samples (Figure 2e,f) exhibit more homogenous and completely coated layers thanks to the functional groups introduced by plasma treatment. In addition to the antithrombotic activities of anticoagulants, the bonding type is also crucial for further interactions with the blood, which is discussed in Section 2.6 in this paper.

### 2.3. Atomic Force Microscopy Characterization

**Figure 3:**
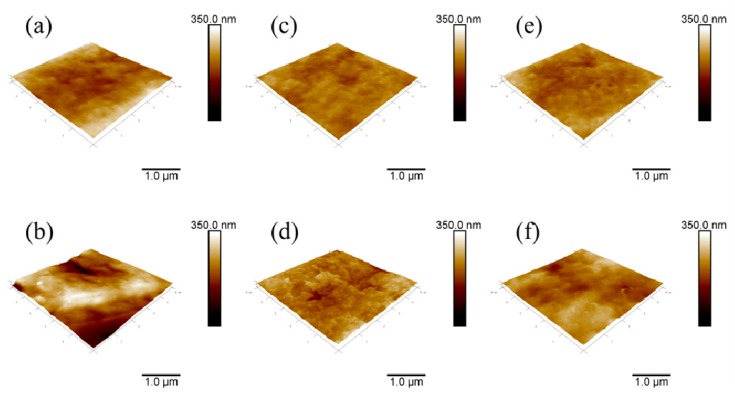
Surface topography by atomic force microscope (AFM): (**a**) PE; (**b**) PET; (**c**) PEH; (**d**) PEF; (**e**) PETH; and (**f**) PETF.

Surface topography investigations of the samples were carried out by AFM; the corresponding images are shown in Figure 3. The surface roughness of the reference PE sample is rather smooth with an average roughness value of 24.2 nm (Table 1). Direct immobilizations of the anticoagulants heparin and fucoidan onto the PE surface decreased the surface roughness to 13.9 and 20.3 nm, respectively (Figure 3c,d). This indicates that heparin and fucoidan particles slightly covered the pores of the PE with a non-uniform feature. However, the PE surface became more rough (46.7 nm) after plasma treatment by etching of the surface (Figure 3b). Similar behavior was observed after the heparin and fucoidan immobilization for PETH and PETF samples in that the layers of anticoagulants decreased the roughness of PET to 17.9 and 24 nm, respectively. The roughness of the PETH and PETF is slightly higher than their PEH and PEF counterparts, which indicates the effect of plasma treatment. It should be emphasized that roughness value itself is not an indicator of the uniformity of the coated layer, and that features of the immobilized anticoagulants should also be take into consideration. Moreover, although the reference PE and PETF show similar surface roughness values, their wettability behaviors are completely different, as was discussed previously in Section 2.1. Therefore, it is evident that wettability, even in the same roughness conditions, is also related to chemical features of the examined substrates. Roughness is an important feature by means of contact area for cell and blood protein adhesion.

### 2.4. Fourier Transform Infrared Spectra Investigations

**Figure 4:**
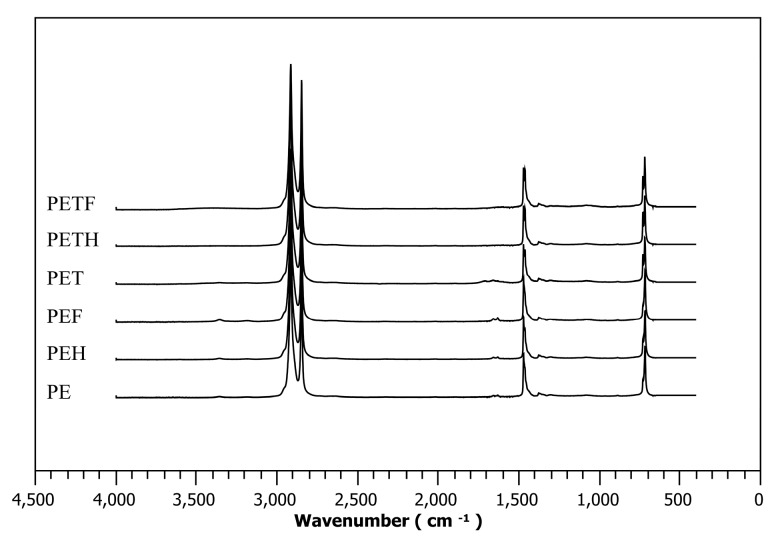
Attenuated total reflectance Fourier transform infrared ATR-FTIR spectra.

Figure 4 shows chemical changes in the near-surface area of the selected samples obtained by attenuated total reflectance Fourier transform infrared (ATR-FTIR) spectra. Regarding the spectrum of the PE, main peaks at the wavenumbers of 2915 and 2850 cm^−1^, which are ascribed to C–H stretching, are visible (typical for aliphatic hydrocarbon chain) [41]. Characteristic signals of C–H bending deformation and methylene rocking deformation appeared at the wavenumbers of 1470 and 719 cm^−1^, respectively. By means of the PET sample, in addition to PE peaks, amine groups introduced by plasma treatment are clearly seen at the wavenumber of 1630 cm^−1^ and oxygen containing hydroperoxides are apparent at the wavenumber of 3370 cm^−1^ [21]. PEH and PEF spectra did not show significant changes to the peaks in comparison to the PET peaks (which means that the peaks at the 3370 and 1630 cm^−1^ are still visible), which serves as evidence for the limited immobilization (or no immobilization at all, but some seen on SEM images) of the heparin and fucoidan on the PE surface without plasma treatment. In the case of PETH and PETF samples, hydroperoxide and amine group peaks are not visible anymore, which serves as an indicator of heparin and fucoidan immobilization on the PE surface after plasma treatment.

### 2.5. X-ray Photoelectron Spectroscopy Investigations

Chemical surface compositions are listed in Table 2. Samples without plasma treatment (PE, PEH, PEF) displayed the same carbon, oxygen, nitrogen, and sulfur levels in regards to the XPS spectra, irrelevant of their exposure to anticoagulants. Constant carbon levels indicate that immobilization of heparin/fucoidan did not take place on the PE surface, or that it took place on a limited basis, since no apparent carbon consumption occurred by introducing of anticoagulants, as we have witnessed partially heparin/fucoidan layers on the surfaces by SEM in Figure 2. As expected, the oxygen level is also constant, which means none of the oxide groups were introduced by plasma treatment yet, and the absence of nitrogen is evidence of free amine groups.

After plasma treatment, the carbon level decreased by about 12% and the oxygen content significantly increased due to the introduced oxides groups; the appearance of a nitrogen level of 1.1% indicates the introduction of amine groups by the plasma treatment.

Anticoagulant immobilization after plasma treatment, in the case of PETH and PETF, decreased the carbon and nitrogen level (amine groups) by chemical bonding and increased the oxygen content due to the oxide containing functional groups present in heparin and fucoidan. Besides consumption of carbon and nitrogen, existing pH levels are the most important evidence of the immobilization of anticoagulants. The immobilized fucoidan layer on the PET sample is greater than that of the immobilized heparin layer, in considering the pH levels (see Table 2). Therefore, it has a more significant impact on antithrombotic properties.

### 2.6. Anticoagulation Activity Studied in Vitro

The blood coagulation cascade consists of the tissue-mediated extrinsic pathway, surface-mediated intrinsic pathway, and common coagulation pathway [42]. The examination of those coagulation pathways were carried out for all samples by means of prothrombin time (PT), activated partial thromboplastin time (aPTT), and thrombin time (TT).

PT assay measures the clot formation time in both the extrinsic and common coagulation pathways. The normal time range for healthy donors is generally between 11–13.5 s [41]. As summarized in Table 3, all samples are within this range.

An aPTT assay is related to both the intrinsic and common coagulation pathways. The normal time range of aPTT for healthy donors is between 25 and 32 s [43]. As summarized in Table 3, none of the samples performed anticoagulant activity on the intrinsic pathway since none of them prolonged aPTT, which indicates no remarkable inhibition of the intrinsic pathway factors. Although, some of the samples exhibited slightly lower aPTT values than the control value, it can be ignored since the differences were still under the physiological threshold.

TT is a measure of thrombin formation time by converting fibrinogen to fibrin in the common coagulation pathway [42]. The lower limit for TT to perform anticoagulation activity is 20 s [43]. The TT of the control PE value (15.9 s) is comparable to the TT value of the plasma modified sample (PET); that indicates plasma induced oxidative functional groups and its increased hydrophilicity were not influencing TT, which is in contrast with the fact that hydrophilicity affects the coagulation cascade, but it is worth noting that pH values and the surface charges are another two crucial parameters to affect the coagulation cascade. Similar results were observed for AAM, MAAM, and DAAM grafted samples (PETA, PETM, and PETD, respectively) with their anticoagulant immobilized counterparts. Antithrombotic activity was not monitored (Table 3) since intramolecular interactions between grafted polymer brushes and anticoagulants were not sufficient for immobilization, which is in agreement with the SEM investigations.

Immobilizations of anticoagulants heparin and fucoidan without plasma treatment (PEH and PEF) did not affect TT due to insufficient immobilization, as was observed in Figure 4. This is consistent with the XPS results in that the PEH and PEF samples did not possess sulfur content in order to increase heparin cofactor II (HCII) based antithrombotic activity [25,39]. Due to the fact that sulfur content was observed only for heparin and fucoidan immobilized samples after plasma treatment (PETH and PETF), expected antithrombotic activity was observed only in the case of the PETF with a TT value of 20.9 s. First and foremost, its sulfur content was 2.4%, while PETH's was only 0.2%. Secondly, the fucoidan immobilization on the PET surface was sufficiently homogeneous in contrast to the heparin immobilized PETH sample, as was observed in Figure 4, therefore its anticoagulation activity was increased; moreover, heparin is covalently bonded and did not interact sufficiently with the coagulation factors.

## 3. Materials and Methods

### 3.1. Materials and Preparation

**Figure 5:**
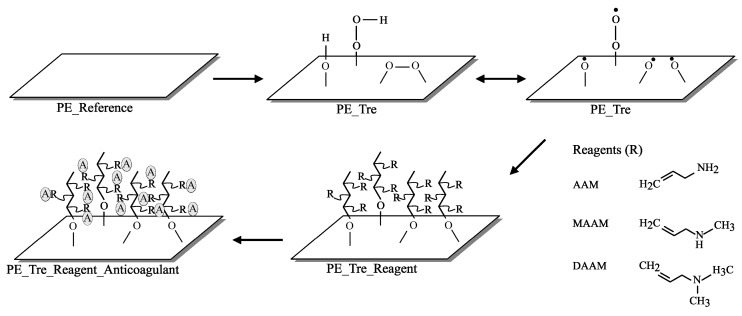
The schematic representation of the process. R stands for grafted Allylamine (AAM), *N*-allylmethylamine (MAAM), and *N*,*N*-dimethylallylamine (DAAM) and A stands for anticoagulants of heparin and fucoidan.

Cylindrical, low density polyethylene (LDPE) Vacuette blood collection tubes (with 20 cm^2^ of contacted surface area) and 4 mL of coagulation sodium citrate (3.2%) were purchased from Greiner Bio-One Company (Kremsmunster, Austria) to use for the anticoagulation activity observation. All blood collection tubes were thoroughly cleaned with distilled water and dried at 30 °C for 24 h in an oven. LDPE film with 100 μm thickness was used as a control substrate to LDPE blood collection tubes. The foil was cut into the form of square flat sheets with the dimension of 50 mm × 50 mm and was used as received without further purification. Both the sheet and tube form of prepared LDPE samples hereafter referred to as PE. Monomers (see Figure 5) of Allylamine (AAM), *N*-allylmethylamine (MAAM) with anticoagulants Fucoidan from *Fucus vesiculosus*, and heparin sodium salt from porcine intestinal mucosa were purchased from Sigma Aldrich (St. Louis, MO, USA) and *N*,*N*-dimethylallylamine (DAAM) was supplied by Fluka (St. Louis, MO, USA). Heparin and fucoidan solutions were prepared as 1% (*w*/*v*) in distilled water and placed in the vials for further use.

### 3.2. Plasma Surface Modification and Reagent Immobilization

The direct current (DC) plasma discharge used for all experiments was generated at the frequency of 40 kHz and power of 50 Watts using a PICO (Diener, Ebhausen, Germany) plasma reactor with a volume of 3 dm^3^. Air was used as a discharge gas with 20 standard cubic centimeter per minute (sccm) flow rate and the pressure in the vacuum chamber was 50 Pa. Both sides of each of the PE sheets and each of the blood collection tubes were exposed to the generated non-thermal plasma for the duration of 60 s (hereafter referred to as PET) to create free radicals and metastable reactive species on the surface to act as initiators for further copolymerization reactions.

After exposing the plasma, the samples were taken out of the chamber and immediately subjected to AAM, MAAM, and DAAM vapors for 20 s in order to immobilize them via a radical graft polymerization process to create functional amine groups containing polymer brushes on the surface, as shown in Figure 5. These samples are hereafter referred to as PETA, PETM, and PETD, respectively. The monomer contact with the radical within its lifetime is of a paramount importance. In our case, we expect the reaction of the monomer with peroxy radicals which have lifetimes in the range of a few seconds [44].

Each PETA, PETM, and PETD substrate were separately placed into heparin/fucoidan containing solution vials and blood collection tubes had been filled up by the same solution and placed in the rotational shaker for 24 h at room temperature in order to immobilize the anticoagulants to the grafted polymer brushes by intramolecular interactions. After 24 h of reaction time, the substrates were taken out of the vials and gently cleaned in water, then distilled water, to eliminate non-immobilized heparin/fucoidan species. In the case of the cylindrical test samples, heparin/fucoidan solutions were poured out and then cleaned carefully by water and distilled water. H and F, which were added in as the last letter of the previous sample abbreviations stand for the heparin and fucoidan immobilized conditions, respectively.

Finally, all samples were dried for 2 h at room temperature. Blood collection tubes were used for anticoagulant activity tests and square flat sheets were used for further tests.

### 3.3. Surface Wettability Evaluation

The sessile drop method was used to evaluate the total free surface energy of the samples. We used the so-called (SEE System) surface energy evaluation system (Advex Instruments, Brno, Czech Republic) equipped with a CCD camera. Distilled water was used as the testing liquid at 22 °C and 60% relative humidity. The volume of the droplets for each experiment was 5 μL and the droplets were kept for 30 s in order to obtain an equilibrium state prior to the measurement. Ten separate readings were averaged to obtain the representative contact angle value in order to estimate the surface hydrophilicity.

### 3.4. Scanning Electron Microscopy

The surface morphology of all samples was observed by scanning electron microscope (SEM) using a NANOSEM 450 (FEI, Hillsboro, OR, USA) performed at 5 kV. The instrument was equipped with a so-called low vacuum detector (LVD), and the measurement was performed in a water vapor environment under 90 Pa pressure with a spot size of 50 nm.

### 3.5. Atomic Force Microscopy

Surface topology characteristics on the samples were obtained by atomic force microscope (AFM) using a Dimension Icon (Bruker, Karlsruhe, Germany). All experiments were performed by peak force tapping mode using a ScanAsyst-Air Si/Nitride probe (Bruker, Santa Barbara, CA, USA) with k = 0.4 N/m of spring constant value of the cantilever. A scanning area of 5 × 5 μm for each sample was investigated with a frequency of 1 Hz. Average surface roughness (*R*_a_) values were analyzed by using NanoScope Analysis software.

### 3.6. Fourier Transform Infrared Spectroscopy

For surface chemistry examination, in order to compare the changes in chemical compositions of the studied samples, a Nicolet iS5 (Thermo Scientific, Grand Island, NY, USA) single beam Fourier transform infrared spectroscopy (FTIR) equipped with iD5 attenuated total reflectance (ATR) was used. Spectra were recorded between 400 and 4000 cm^−1^ with a resolution of 2 cm^−1^ and 64 scans using a ZnSe crystal at an incident angle of 45°.

### 3.7. X-ray Photoelectron Spectroscopy

The chemical composition of the surfaces was analyzed with X-ray photoelectron spectroscopy (XPS) using TFA XPS (Physical Electronics, Chanhassen, MN, USA). The samples were exposed to X-rays over a 400 μm spot size with a monochromatic Al K_α1,2_ radiation at 1486.6 eV, under 6 × 10^−8^ Pa chamber pressure. The emitted photoelectrons were detected with a hemispherical analyzer placed at an angle of 45° in order to correlate to the normal plane of the samples. Survey-scan spectra were made with a 0.4 eV step resolution at 187.85 eV of pass energy. Surface neutralization was carried out by an electron gun, and MultiPak (Version 7.3.1) software (Physical Electronics, Chanhassen, MN, USA) was used to analyze elemental concentration.

### 3.8. Anticoagulation Activity Test

For anticoagulation tests, the blood was obtained by venous puncture from a healthy donor in accordance with the Helsinki Declaration, and was placed into blood collection tubes (5 mL each). The obtained human blood plasma was treated with 3.2% citric acid (109 mmol/L) and then centrifuged at 3000× *g* for 15 min at room temperature. Anticoagulant activity was determined by means of prothrombin time (PT), thrombin time (TT), and activated partial thromboplastin time (aPTT) using a SYSMEXCA—1500 (Siemens, Munich, Germany) instrument. Each of the samples was examined three times.

## 4. Conclusions

Plasma treatment onto LDPE and its effect on further anticoagulant heparin and fucoidan immobilizations has been studied. Surface wettability remarkably increased as a result of the introduced oxidative functional groups by plasma treatment and surface roughness increased by simultaneous etching, as was revealed by both the contact angle measurement and AFM. Anticoagulant immobilization on the LDPE surface without plasma treatment was not sufficient, as it observed from SEM images and XPS results, but treated surfaces showed more homogeneous layers and sulfur content; notably, fucoidan immobilization was more successful than heparin. Anticoagulation tests revealed that the PETF sample has anticoagulation effects, as evidenced by its more homogeneous immobilization and also the higher anticoagulation effect of fucoidan in comparison to heparin. Hence, surface modification of LDPE by plasma treatment followed by fucoidan immobilization represents an effective route to prevent surface mediated thrombus formations of blood contacting biomaterial.

## Figures and Tables

**Table 1:** Water contact angle (θ_w_) and average surface roughness (*R*_a_) values.

Samples	PE	PEH	PEF	PET	PETH	PETF
θw (°)	85.3 (±3.4)	81.9 (±12.2)	79.4 (±8.3)	56.9 (±11)	79.6 (±5.3)	59.1 (±9)
*R*_a_ (nm)	24.2	13.9	20.3	46.7	17.9	24

PE, reference polyethylene samle; PEH, heparin immobilized sample before plasma treatment; PEF, fucoidan immobilized sample before plasma treatment; PET, plasma modified sample; PETH, heparin immobilized sample after plasma treatment; PETF, fucoidan immobilized sample after plasma treatment.

**Table 2:** Elemental compositions and ratios of the tested surfaces obtained by X-Ray photoelectron spectroscopy (XPS).

Samples	C1s%	O1s%	N1s%	S2p%	O1s/C1s	N1s/C1s	S2p/C1s
PE	98.7	1.3	-	-	0.013	-	-
PEH	98.6	1.4	-	-	0.014	-	-
PEF	98.7	1.3	-	-	0.013	-	-
PET	87.2	11.7	1.1	-	0.134	0.013	-
PETH	85.7	13.3	0.8	0.2	0.155	0.009	0.0023
PETF	82.3	14.8	0.5	2.4	0.18	0.006	0.0292

**Table 3:** Antithrombotic test results. PT: Prothrombin time; aPTT: activated partial thromboplastin time; TT: Thrombin time.

Samples	PT (S)	aPTT (S)	TT (S)
PE (Reference)	11.5	24.8	15.9
PEH	11.5	25	16.5
PEF	11.2	24.1	16.8
PET	11.4	25.9	16.8
PETH	11.5	25.7	16.6
PETF	10.9	27.3	20.9
PETA	11.5	25.2	16.3
PETAH	11.6	26.5	17.2
PETAF	11.1	26.5	18
PETM	11.7	26.4	16.9
PETMH	11.4	25.2	16.7
PETMF	11	24.2	16.5
PETD	11.4	24.2	16.6
PETDH	11.4	24.1	16.5
PETDF	11	27.1	19.5
